# Supplementary material for: Causal Relationship between Aging and Anorexia Nervosa: A White-Matter-Microstructure-Mediated Mendelian Randomization Analysis
Source: Biomedicines. 2024 Aug 16;12(8):1874. doi: 10.3390/biomedicines12081874 (PMC11351342; doi:10.3390/biomedicines12081874)
Supplement: Supplementary file 1 [file biomedicines-12-01874-s001.zip › Supplementary Figure/Figure S4/Singe SNP plot of MR analysis for TL to ubm-b-2063.pdf]

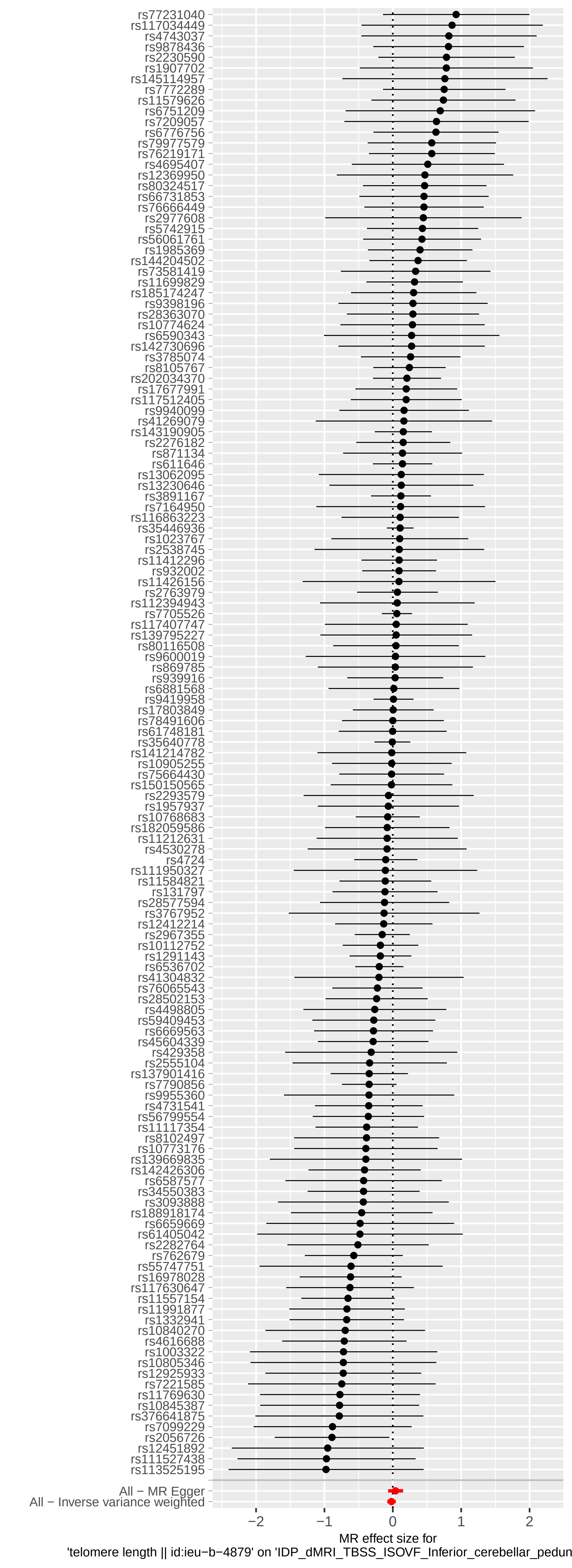

rs77231040
rs117034449
rs4743037
rs9878436
rs2230590
rs1907702
rs145114957
rs7772289
rs11579626
rs6751209
rs7209057
rs6776756
rs79977579
rs76219171
rs4695407
rs12369950
rs80324517
rs66731853
rs76666449
rs2977608
rs5742915
rs56061761
rs1985369
rs144204502
rs73581419
rs11699829
rs185174247
rs9398196
rs28363070
rs10774624
rs6590343
rs142730696
rs3785074
rs8105767
rs202034370
rs17677991
rs117512405
rs9940099
rs41269079
rs143190905
rs2276182
rs871134
rs611646
rs13062095
rs13230646
rs3891167
rs7164950
rs116863223
rs35446936
rs1023767
rs2538745
rs11412296
rs932002
rs11426156
rs2763979
rs112394943
rs7705526
rs117407747
rs139795227
rs80116508
rs9600019
rs869785
rs939916
rs6881568
rs9419958
rs17803849
rs78491606
rs61748181
rs35640778
rs141214782
rs10905255
rs75664430
rs150150565
rs2293579
rs1957937
rs10768683
rs182059586
rs11212631
rs4530278
rs4724
rs111950327
rs11584821
rs131797
rs28577594
rs3767952
rs12412214
rs2967355
rs10112752
rs1291143
rs6536702
rs41304832
rs76065543
rs28502153
rs4498805
rs59409453
rs6669563
rs45604339
rs429358
rs2555104
rs137901416
rs7790856
rs9955360
rs4731541
rs56799554
rs11117354
rs8102497
rs10773176
rs139669835
rs142426306
rs6587577
rs34550383
rs3093888
rs188918174
rs6659669
rs61405042
rs2282764
rs762679
rs55747751
rs16978028
rs117630647
rs11557154
rs11991877
rs1332941
rs10840270
rs4616688
rs1003322
rs10805346
rs12925933
rs7221585
rs11769630
rs10845387
rs376641875
rs7099229
rs2056726
rs12451892
rs111527438
rs113525195
All − MR Egger
All − Inverse variance weighted
−2
−1
0
1
2
MR effect size for
'telomere length || id:ieu−b−4879' on 'IDP_dMRI_TBSS_ISOVF_Inferior_cerebellar_pedun
